# Skeletal Aging and Osteoporosis: Mechanisms and Therapeutics

**DOI:** 10.3390/ijms22073553

**Published:** 2021-03-29

**Authors:** Abhishek Chandra, Jyotika Rajawat

**Affiliations:** 1 Department of Physiology and Biomedical Engineering, Mayo Clinic, Rochester, MN 55902, USA; 2 Department of Internal Medicine, Division of Geriatric Medicine and Gerontology, Mayo Clinic, Rochester, MN 55902, USA; 3 Robert and Arlene Kogod Aging Center, Mayo Clinic, Rochester, MN 55902, USA; 4 Department of Zoology, University of Lucknow, University Rd, Babuganj, Hasanganj, Lucknow, Uttar Pradesh 226007, India; jrajawat@gmail.com

**Keywords:** osteoporosis, senescence, SASP, aging, radiation, senotherapeutic

## Abstract

Bone is a dynamic organ maintained by tightly regulated mechanisms. With old age, bone homeostasis, which is maintained by an intricate balance between bone formation and bone resorption, undergoes deregulation. Oxidative stress-induced DNA damage, cellular apoptosis, and cellular senescence are all responsible for this tissue dysfunction and the imbalance in the bone homeostasis. These cellular mechanisms have become a target for therapeutics to treat age-related osteoporosis. Genetic mouse models have shown the importance of senescent cell clearance in alleviating age-related osteoporosis. Furthermore, we and others have shown that targeting cellular senescence pharmacologically was an effective tool to alleviate age- and radiation-induced osteoporosis. Senescent cells also have an altered secretome known as the senescence associated secretory phenotype (SASP), which may have autocrine, paracrine, or endocrine function. The current review discusses the current and potential pathways which lead to a senescence profile in an aged skeleton and how bone homeostasis is affected during age-related osteoporosis. The review has also discussed existing therapeutics for the treatment of osteoporosis and rationalizes for novel therapeutic options based on cellular senescence and the SASP as an underlying pathogenesis of an aging bone.

## 1. Introduction

Bone as a tissue has its own complexities with one of the largest pools of diverse cell types. Consisting of inorganic ions (30%) and collagenous and non-collagenous proteins (70%), bone also serves as a reservoir for minerals such as calcium, phosphorous, magnesium, sodium, and bicarbonate. Together with tight regulations from hormones such as Vitamin D (cholecalciferol), parathyroid hormone (PTH), and calcitonin, and supported by several organ systems, these minerals are maintained in a homeostasis. This perfect harmony of minerals is supported by various cells in the bone compartment, which may have altered functions due to a familial genetic alteration (associated with mechanisms underlying primary osteoporosis), hormonal changes (loss of estrogen in post-menopausal women, and loss of androgens in men), physiological aging, and pathological changes (disease or treatment related), leading to other forms of osteoporosis.

Physiological aging is often linked with several life altering co-morbidities, osteoporosis being one of the prevalent among them. Mechanisms underlying age-related osteoporosis are partially understood, and often misunderstood with estrogen-deprivation related osteoporosis seen in post-menopausal women.

**Figure 1:**
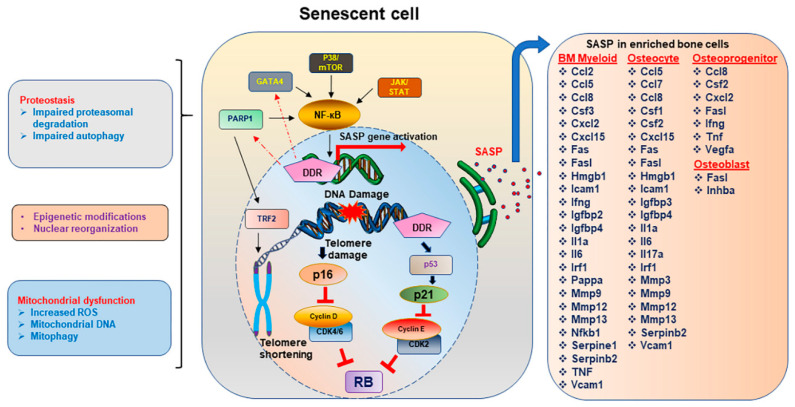
Spectrum of changes in a senescent cell. DNA damage response (DDR) is one of the key inducers of cellular senescence, and if the DNA damage is in the telomere sites, this drives the cell towards a senescent state which has several characteristics, also acting as sustainers or inducers of the senescent state of the cell. Telomere shortening or damage driven DDR initiates the p16^Ink4a^ or p21 driven pathways which block the cyclin D, cyclin dependent kinase (CDK)2/4/6, and cyclin E to thereby stabilizing the retinoblastoma (Rb) protein, allowing the cell to enter the arrest phase. Activation of nuclear factor kappa B (NF-кB) through indirect activation of PARP1, GATA4, p38/ mechanistic target of rapamycin (MTOR), or Janus Kinase/Signal Transducer and Activator of Transcription (JAK/STAT) pathways activate the transcription of senescence associated secretory phenotype (SASP) genes. Proteostasis, either by impairment in the ubiquitin proteasome system or the autophagy pathway, allows aggregation of unwanted proteins, contributing to senescent profile of the cell. Mitochondrial dysfunction, including changes in the mitochondrial DNA, increased reactive oxygen species (ROS) and altered autophagy of the mitochondrial compartments, contributing to the overall stressed environment leading to senescence. Chemokines, interleukins, and matrix modifying enzymes form the bulk of the proinflammatory SASP genes which may work in an autocrine, paracrine, or endocrine manner. The list of SASP proteins was generated based on their expression in enriched bone cells. SASP abbreviations—TRF2: telomeric repeat binding factor (TRF), Ccl: C-C motif chemokine ligand, Csf: colony-stimulating factor, Cxcl: chemokine (C-X-C motif) ligand, HMGB1: high mobility group box 1, Icam1: intercellular adhesion molecule 1, Ifng: interferon gamma, Igfbp: Insulin-like growth factor binding proteins, Il: Interleukin, Irf1: Interferon Regulatory Factor 1, Mmp: Matrix metallopeptidase, Pappa: pregnancy-associated plasma protein A, TNF: Tumor necrosis factor, Vcam1: Vascular Cell Adhesion Molecule 1.

Advent of loss of bone with aging are also early signs of increased fracture risk, morbidity, and mortality. Osteoporotic fractures exceed incidences of cardiovascular disease or cancer by ~3 to 4-fold [1] and is a substantial strain on the economy. Only 31–36% people above the age of 70 have normal bones, while the remainder suffering from some form of osteopenia or osteoporosis. Moreover, it is well understood that loss of estrogen is a key driver of bone loss in women and to some extent in men, which is only exacerbated by aging [2,3]. However, estrogen is not the only cause of bone loss during aging [4], and it has been recognized and discussed in detail that aging stands as a separate entity with distinct mechanisms [5]. This review will expand on known and potential mechanisms underlying the pathogenesis of skeletal aging, mainly DNA damage and cellular senescence, with special emphasis on certain proteins such as poly (adenosine diphosphate (ADP)-ribose) polymerase 1 (PARP1) due to their role in DNA repair, telomere maintenance, senescence, and the production of pro-inflammatory cytokines, the senescence associated secretory phenotype (SASP) (Figure 1).

## 2. Biology of Skeletal Aging

Bone is a dynamic organ incorporating several cell types which generally work synchronously and maintain the bone homeostasis resulting in the deposition of a mineralized bone matrix. The two processes which maintain this homeostasis are bone formation and bone resorption which are under an equilibrium in a normal physiological condition. Cells of the mesenchymal origin regulate the bone formation process including bone marrow stem cells (BMSCs) which are the progenitors to osteoblasts, cells responsible to deposit mineral and form the collagen enriched bone matrix supported by the multifaceted osteocytes, the most abundant cell type with an extensive canalicular network.

**Figure 2:**
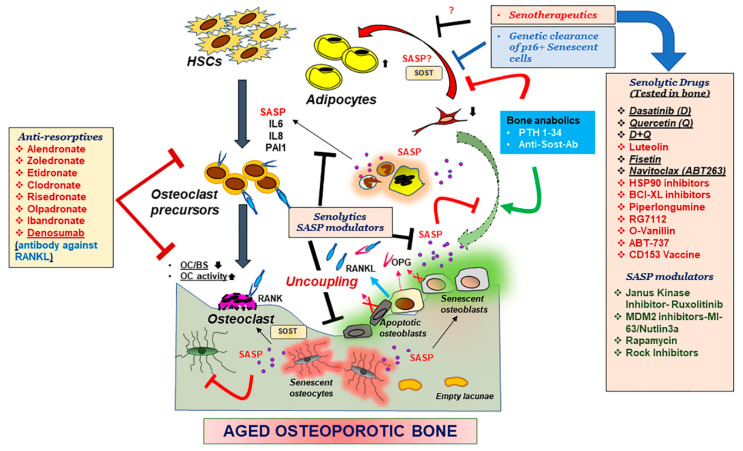
Mechanisms underlying an aging skeleton and potential therapeutic options. Bone formation which entails recruitment of bone marrow stem cells (BMSCs) to the bone surface, differentiation into osteoblasts and mineralization by the osteoblasts is followed by further differentiation of osteoblasts into osteocytes, which embed in the matrix, thereby communicating with other osteocytes, or cells in the bone environment through canalicular networks. Hematopoietic stem cells (HSCs) and precursors to the osteoclasts are activated by the binding of the RANKL to the RANK receptor, promoting osteoclastogenesis and bone resorption. Osteoprotegerin (OPG) a decoy receptor to RANKL, secreted by the osteoblasts, blocks the binding of RANKL to RANK and blocks osteoclastogenesis. With aging, osteoblasts and osteocytes undergo apoptosis or cellular senescence, and in the process, this internal regulation by OPG is disturbed, leading to more resorption. Production of pro-inflammatory SASP exacerbates the suppression of osteoblast function while triggering an activation of osteoclast precursors towards osteoclastogenesis. Moreover, reduction in BMSCs due to an altered fate to adipogenesis, also contributes to the suppression of osteoblast function. Reduction in osteoclast numbers, but increased activity, also disturbs the recruitment of more BMSCs to the bone surface, thus causing uncoupling of the bone homeostasis. Bone anabolics such as PTH 1-34 and neutralizing antibody against sclerostin (Sost), and anti-resorptives as shown in the figure have been effective treatments for post-menopausal osteoporosis, but their efficacy in an aging population is not determined. Genetic removal of senescent cells was shown to restore bone homeostasis in aged mice hence pharmacological targeting senescent cells became a lucrative therapeutic option. Drugs that can remove the senescent cell (Senolytic drugs) or suppress the production of SASP (SASP modulators), collectively called senotherapeutics, may remove the triggers for uncoupling and restore bone homeostasis. Several of these senotherapeutics are listed in the figure and the ones which have been tested in some form of skeletal aging are underlined. Abbreviations- OC: osteoclast, BS: bone surface, Il: interleukin, PAI1: plasminogen activator inhibitor 1.

Osteoclasts, a large multinucleate cell, having a hematopoietic origin, is responsible to resorb the bone matrix and is regulated by extracellular signals secreted by the osteoblasts and osteocytes while being supported by progenitors such as bone marrow monocytes or macrophage precursors [6,7,8]. Differentiation of osteoclasts require binding of receptor activator of nuclear factor kappa-Β (RANK) ligand (RANKL) to the RANK receptor on the osteoclast surface [9,10] together with the secretion of macrophage colony-stimulating factor (M-CSF) by osteoblasts and bone marrow stromal cells [11]. MCSF then activates the proliferation of the osteoclast precursors by binding to the colony stimulating factor-1 receptor (CSF-1R) also known as c-FMS. Parathyroid hormone (PTH), 1,25-vitamin D3, IL-1, IL-6, IL-11, and tumor necrosis factor (TNF) are some of the factors that directly or indirectly influence osteoclast differentiation [12]. Osteoprotegerin (OPG), a humoral tumor necrosis factor (TNF) receptor family protein, secreted by several cell types including osteoblasts, acts as a decoy receptor to RANK blocking the binding of RANKL to RANK (Figure 2). Reduction of osteoprogenitors and osteoblasts with age reduces the OPG levels which in turn allows the activation of osteoclast-based resorption, thus tilting the balance of bone homeostasis, causing osteopenia and osteoporosis. Resorption of bone matrix that include type I collagen, the predominant component of the matrix [13] allows the release of matrix associated proteins such as transforming growth factor β1 (TGF-β1) and Insulin-like growth factor type I (IGF-1), which then promote mesenchymal cell differentiation to form mature osteoblasts [14,15,16,17]. Many such factors secreted by the osteoclasts are known as coupling factors. Osteocytes, the terminally differentiated osteoblasts, were a major source of RANKL thus promoting osteoclastogenesis and an osteocyte specific deletion of RANKL resulted in osteopetrosis [18].

Osteocytes are one of the most abundant cell types in the bone tissue and contribute to sensing mechanical load [19], through their extensive network of lacuna-canalicular area amounting to 215m^2^ [20]. Osteocytes may positively or negatively regulate bone remodeling [21], a dynamic which tilts to a negative regulation with aging during which the lacunar density declines [22].

Age related functional decline in osteoblasts due to increased apoptosis [23], decreased proliferation, impaired osteoblast differentiation [24], increased osteoblast senescence [25] and dysfunctional osteoprogenitors [26], leading to more marrow adipogenesis as the favored pathway [27]. The decline in bone mass with old age is inversely proportional to the bone marrow adipose tissue (BMAT) accumulation. BMAT is also reported in post-menopausal women, due to immobilization as seen in spinal cord injury and with steroid treatments, with age being a synergistic confounder.

Inheritable changes in several genes have been implicated in the pathogenesis of idiopathic osteoporosis and skeletal aging [28]. Several osteoblastic genes such as *WNT10B, RUNX2, RANKL, Osterix, Osteocalcin, OPG,* and *SOST*, were found to be differentially expressed in patients with male idiopathic osteoporosis, characterized with low bone volume and decrease in trabecular number. These changes were attributed to dysfunctional osteoblasts, and reduction in *WNT10B, RUNX2, RANKL,* and *SOST* gene expression [29]. Studies done using RNA-sequencing assessed genes that change with age-related osteoporosis and found ~700 differentially expressed genes and 12 cellular pathways involved in aged bones [30]. Genetic polymorphisms in the *CaSR*, a gene that encodes for the calcium sensing receptor and mainly regulates calcium homeostasis, has been associated with determining the prevalence of osteoporosis in aging males [31]. Certain genetic polymorphisms in the collagen type Ialpha1 gene (*COL1A1*) have been associated with post-menopausal osteoporosis [32,33,34]. In another study, *WNT1* mutations were linked to early onset osteoporosis, high fracture rate and low bone turnover [35]. The study also showed that Wnt1 was a potent bone anabolic agent, independent of the LRP5-associated Wnt-pathway. Role of *WNT1* mutations in age-related osteoporosis is still to be determined.

## 3. Senescence

For many years it was believed that cells in vitro could grow uncontrollably. It was first shown by Hayflick and Moorehead that cells in culture do undergo replicative senescence [36]. Apart from replicative senescence, which is linked to telomere shortening during cell cycle and is linked to organismal aging, cytotoxic stress-induced premature senescence (SIPS) can be triggered by oncogene activation [37], accumulation of free radicals, reactive oxygen species, DNA damage in general and of the telomeres, proteostasis, mitochondrial dysfunction, activation of pro-survival pathways, etc. [38,39]. These changes eventually lead to a cellular morphology which looks enlarged and flattened, with ruffled cellular surfaces [40], increased cellular debris, and often accompanied with chromatin modification, also known as senescence-associated heterochromatin foci (SAHF) [41]. Two signaling pathways, ataxia telangiectasia mutated (ATM)/p53/p21^Cip1^ and p16^INK4a^/RB, regulate the senescence spectrum. DDR initiates the stabilization of tumor suppressor, p53 which in turn induces cyclin dependent kinase (CDK) inhibitor (CDKi), *p21^Cip1^*, which initiates cell cycle arrest [42,43]. *p16^INK4a^* was shown to directly bind and inhibit the catalytic activity of CDK4 [44] and CDK6 [45]. The ultimate result of activation of *p21^Cip1^* or *p16^Ink4a^* and the blockade of D type cyclin and CDK4/6, thereby activating the tumor suppressor retinoblastoma protein (RB). Accumulation of *p16^Ink4a^* has been shown to promote tumor progression and age dependent co-morbidities, and clearance of p16-positive cells starting from mid-life suppressed tumor progression with aging, and other age-related tissue dysfunction [46].

Increased life and health span have been achieved by clearance of senescent cells using pharmacological and genetic mouse models [47,48]. In this review we have discussed the role of key inducers of cellular senescence during the process of skeletal aging.

### 3.1. Inducers of Cellular Senescence and Osteoporosis

Since the early descriptions of cellular senescence, several inducers of senescence have been defined over the decades including telomere dysfunction, DNA damage, chromatin aberrations, reactive oxygen species (ROS), and oncogenes among others.

#### 3.1.1. DNA Damage and Genomic Instability

Accumulation of mutations and DNA damage throughout life is a major factor for aging. DNA is continuously exposed to exogenous as well as endogenous threats leading to genetic lesions. Chemical exposure, physical damage and biological agents are exogenous agents causing DNA damage. Endogenous threats include error prone DNA replication, generation of reactive oxygen species (ROS) and hydrolytic reactions [49]. Genetic lesions caused by these agents lead to compromised genetic integrity and gradually aging. DNA damage triggers a DDR response which is regulated by several pathways. A damaged DNA may undergo: (i) simple reversal in an error-free manner capable of fixing simple alkylated bases, (ii) a base excision repair (BER) attends to oxidative, deamination, alkylation, and abasic single base damage, (iii) nucleotide excision repair (NER) addressing bulky base repairs, (iv) mismatch repair (MMR) which maintains the replicative fidelity, (v) inter cross-link repair (ICL) fixes covalent linkages of adjacent DNA strands, and (vi) the DNA break repair which include single strand break (SSB) repair and double strand break (DSB) repair.

Key proteins of these pathways are also used to often define DDR pathways such as, ATM kinase, ataxia telangiectasia and Rad3 related (ATR) kinase, PARP1] and three DSB repair pathways [classical nonhomologous end joining (c-NHEJ), alternative (alt)-NHEJ, and homology-directed repair (HDR)].

One of the earliest reports showing accelerated aging caused due to a mutation in Xeroderma pigmentosum (XP)-type D (XPD), a gene encoding a DNA helicase that functions in both repair and transcription. Mutation in this gene resulted in a human disorder trichothiodystrophy (TTD). TTD mice were found to exhibit many symptoms of premature aging, including osteoporosis [50]. Reduced bone mass was observed in ATM kinase deficient mice with defects on osteoblast differentiation and increase in osteoclastogenesis [51]. ATM-/- mice also reported reduced osterix protein levels in the calvarial osteoblasts. A similar reduction in bone mass was observed in an inducible deletion of ATR kinase, together with other premature aging phenotypes [52]. Excision repair cross complementary group 1–xeroderma pigmentosum group F (ERCC1-XPF) is an endonuclease that plays a role in several DNA repair pathways. Genetic mutations in the ERCC1-XPF gene in humans have been shown to have progeria like state with osteoporosis as one of the phenotypic pathologies. *Ercc1*-null and hypomorphic mice both displayed severe osteoporosis, with bone resorption outpacing bone formation [53]. These mice also displayed increased cellular senescence and SASP, which was reduced by downregulating the nuclear factor kappa B (NF-κB) [53].

Exogenous DNA damage caused by ionizing radiation (IR) has also been shown to be partly responsible in reduction of bone forming cells in mice. Anabolic agents such as PTH 1–34 and neutralizing antibody against sclerostin (Scl-Ab) and anti-resorptive drug zoledronate have been shown to counter DNA damage seen in radiated bones or BMSCs [54,55,56]. Stabilization of DNA repair proteins Ku70 and DNA-PKC were also shown to protect osteoporosis in radiated bones [57].

#### 3.1.2. Telomere Dysfunction

Telomeres are repeated DNA sequences of TTAGGG and may comprise up to a thousand repeats located at the end of the chromosome forming a cap of proteins. Telomeres serve as a molecular clock and maintain the replicative potential of a cell. Exhaustion of telomeres is a major factor of normal aging and with each cell division there is shortening of the telomere length [58,59]. Apart from replicative senescence, telomere damage due to oxidative stress can also lead to cellular senescence. The damaged telomere is identified as a DSB and initiates a DDR [60]. Recruitment of DDR pathway proteins follows the initial triggers and colocalization of DDR proteins to the telomere have been successfully used to identify dysfunctional telomeres in aged- and radiated bones, often defined by different acronyms such as Telomere dysfunction-induced foci (TIF) or Telomere associated foci (TAF) [25,61]. These events trigger the activation of *p53/p21^Cip1^* and *p16^Ink4a^* senescent pathways which ceases the growth of the cell [62].

Two human genetic diseases namely Werner’s syndrome (WS) and dyskeratosis congenita, with premature aging symptoms such as osteoporosis [63], were confirmed in an accelerated model of aging in mice where WS helicase and telomerase were genetically removed [64]. It was later reported that single mutation in the telomerase gene (*Terc*) and double mutants of WS helicase and telomerase (*Wern-/-Terc-/-*) showed accelerated age-associated osteoporosis [65]. Mutations in the genes associated with the telomerase complex (dyskerin, TERC, TERT, NHP2, and NOP10) are also associated with dyskeratosis congenita, an accelerated aging syndrome, characterized by greying, dental loss, osteoporosis, and malignancy [66]. These studies suggest that maintenance of the telomere function is key in the overall aging process, including osteoporosis.

#### 3.1.3. Epigenetic Alterations

Defined as heritable changes in the gene expression and independent of the changes in the DNA sequence itself, several “epigenetic” alterations include DNA methylation, histone modifications and non-coding RNA, are associated with aging and aging-associated comorbidities. Epigenetic changes comprise of a complex set of cellular processes and are also used as predictors of bone loss with aging.

DNA methylation in general declines with aging. Osteoporosis and osteoarthritis were correlated with methylation levels at CpG loci in aged women [67]. An epigenome-wide association study (EWAS) of BMD was performed looking across ~500,000 CpGs quantified in whole blood from ~4500 patients but failed to identify DNA methylation sites as a reliable predictor of BMD when assessed in peripheral blood [67]. Another study confirmed that detecting DNA methylation in blood was not found to be a good sample type as a predictor for osteoporosis in aged patients [68]. One study which detected hypomethylation of the Alu elements in the blood cells of women with post-menopausal osteoporosis, associated Alu hypomethylation with other age-associated comorbidities as well [69]. A better correlation between CpG methylation was achieved when comparisons were made between blood and bone biopsies from post-menopausal women with osteoporosis [70]. Based on the available literature, there was a consensus that further studies are required to make direct correlation between DNA methylation patterns and skeletal aging.

Some of the major modification of histones can happen either by methylation (addition of methyl groups by histone methyl transferases, HMTs), demethylation (histone demethylase), acetylation (transfer of acetyl group by histone acetyl transferase, HATs), deacetylation (histone deacetylases, HDACs), or phosphorylation (occurs at serine, threonine, and tyrosine residues, controlled by histone kinases and phosphatases). Apart from these deamination (conversion of arginine to citrulline, by peptidyl deiminase), ubiquitylation and sumoylation and the less studied ADP ribosylation.

HDACs catalytically hydrolyze the N6-acetyl-lysine residues of histones, thereby deacetylating the substrate protein. HDAC1 and HDAC3 are one of the highly produced HDACs in adult human bone and articular cartilage. HDAC7 has been shown to suppress osteoclastogenesis [71]. Conversely, Sirtuin 1 (Sirt1), another histone deacetylase is linked with maintenance of osteoblast progenitor proliferation and survival, which declines with age [72]. HDAC inhibitors (HDACi) have been shown to elevate osteoblast maturation through a Runx2 dependent pathway [73].

Trimethylation of the histone H3 at lysine 27 (H3K27) by histone methyltransferase EZH2 has been shown to regulate osteogenesis [74,75,76,77]. A genome-wide methylation analysis among osteoporotic and osteoarthritic populations identified unique methylation sites, suggesting a role of epigenetic regulation in the two bone pathologies [78]. A histone lysine-specific demethylase, LSD1 has been reported to be essential for endochondral ossification during fracture healing [79]. LSD1 also regulates bone accrual by controlling the expression of Wnt7b and BMP2 [80]. Histone demethylases KDM4B and KDM6B have been shown to promote osteogenesis of human MSCs [81].

Several chromatin modifying enzymes such as HAT1, KAT5, HDAC6, MBD1, and DNMT3A were downregulated at gene expression level in women with post-menopausal osteoporosis and osteoarthritis, with superior quantity and quality of bone being directly associated with *HAT1*, *HDAC6*, and *MBD1* expression [82]. H3K9 acetyltransferase PCAF was found to play a key role during osteogenic differentiation of BMSCs [83].

Micro-RNAs (miRNAs, ~22 nucleotides long) and long non-coding RNAs (~200 nucleotides long) also play a key role in the process of bone remodeling and aging often by directly regulating the senescence pathway. Several miRNA’s have been implicated in the process of age- or post-menopausal osteoporosis. miRNA-148a-3p was found to be elevated in plasma of osteoporotic patients [84,85]. It is interesting to note that miRNA-195, which is a negative regulator of telomerase reverse transcriptase (TERT) and increase with age [86], regulates BMSC senescence [86] and is also implicated in the osteogenic differentiation process [87]. miRNA-219a-5p is another miRNA which has direct role in regulating osteogenic differentiation during aging by directly targeting orphan nuclear receptor, Rorβ [88]. miRNA-182 was reported to negatively regulate osteoblast proliferation and differentiation by targeting FoxO1 [89]. miRNA-19a-3p was shown to be down-regulated in osteoporotic patients, and overexpression of miRNA-19a-3p downregulated HDAC4 and upregulated the *RUNX2* and *OCN.* levels [90]. LncRNA TERC is present in low levels when tested in patients with osteoporosis and has been shown to regulate osteogenic differentiation by absorbing miRNA-217 by upregulating RUNX2 [91]. Conversely, certain miRNA, such as miRNA-483-5p [92], miRNA-133a [93], miRNA-17 [94], and miRNA 199a-5p [95], which promote osteoclastogenesis are also implicated in the pathogenesis of osteoporosis.

In an attempt to establish circulatory miRNAs as a biomarker signature, samples analyzed from premenopausal, post-menopausal women with osteoporosis and men, all with fragility fractures, significant correlations were reported between miRNA-29b-3p and P1NP, miRNA-365-5p and iPTH, TRAP5b, P1NP and Osteocalcin, and finally between BMD and miR-19b-3p, miR-324-3p, miR-532-5p, and miR-93-5p [96]. Among these, miRNA-29b-3p and miRNA-324-3p were also found to be correlated to bone architecture in another study looking for circulatory miRNAs [97]. miR-29 family have been reported as positive regulators of osteoblast differentiation in several studies [98,99], with reports suggesting miR-29b-3p positively regulating femoral fracture healing preclinically [100]. The studies with miRNAs should be seen with certain caveats, one of them is the multitude of targets which these miRNAs influence and the crosstalk between different organ systems in which miRNAs may be targeting different gene functions.

#### 3.1.4. Loss of Proteostasis

Aggregated or misfolded proteins are known to induce age related disorders like, Parkinson’s and Alzheimer’s diseases. Accumulation of proteins occur due to a dysfunction of the cellular machinery which breaks down proteins, shared between autophagy and the 26S-proteasome system. A reduction in autophagy causes loss of proteostasis leading to cellular senescence [101]. A genetic deletion of the autophagy related 7 (ATG7), a key component of the autophagy machinery, showed deterioration in bone mass [102]. In another study autophagy inhibitor 3-methyladenine made BMSCs senescent reducing their osteogenic ability, while autophagy induced rapamycin could restore bone mass in aged mice [103].

In some other cell types, proteasome inhibition induces senescence [104,105,106]. Proteasome inhibitors are successful therapeutics for treatment of multiple myeloma and negatively affect cancer cell growth. While proteasome function is important during aging and any reduction in function leads to senescence, this story is not without caveats. Based on our work and others, proteasome inhibition improves osteoblast function and improves bone formation, while suppressing osteoclast-based resorption and suppressing proteasome function at least by certain inhibitors, has anabolic effects on bone formation [57,107,108]. It was shown that higher the metabolic activity of a cell, the more susceptible the cell is to undergo senescence due to proteasome inhibition, while quiescent cells are shown to be resistant to proteasome inhibitor induced toxicity [109]. Endogenous proteasome suppression during aging does result in senescence, and in bone loss. This can be attributed to the cumulative cellular events such as impaired autophagy, mitochondrial dysfunction, and impaired endoplasmic reticulum.

#### 3.1.5. Mitochondria and ROS

Being the cellular powerhouse, mitochondria utilize the maximum intracellular oxygen, while producing energy and generating ROS in the process. ROS produced by mitochondria in turn causes DSB in the DNA and activates the DDR. Oxidative stress has been a known inducer of senescence shown in cells grown in high oxygen concentration [110]. It was recently reported that in the absence of mitochondria, senescent cells had reduced ROS, reduced cytoplasmic chromatin fragmentation and a reduced pro-inflammatory SASP profile [111]. Low levels of ROS can maintain bone homeostasis and a balance between osteoblasts and osteoclasts [112]. Abnormal levels of ROS have been shown to cause cell death in osteoblasts and osteocytes and reduction in bone architecture [113]. Increase in ROS and a reciprocal decrease in antioxidant levels accounts for an elevated osteoclast activity and reduced osteogenic potential of osteoblasts causes bone deterioration as seen in human studies [112,114,115]. Osteoclasts are multinucleated cells, and thus have a high energy requirement provided by the mitochondria, which helps in the acid production during bone resorption.

Mitochondrial DNA is another focus of research in aging and its associated comorbidities. mtDNA polymerase gamma (Polg), a lone DNA polymerase found in mitochondria, when mutated, showed accelerated age-related osteoporosis with reduced osteogenic potential and increased osteoclasts activity [116].

## 4. Cellular Senescence and Skeletal Aging

The earliest studies that defined the role of cellular senescence in bone deterioration came in the senescence accelerated mice (SAM-R/3 and SAM-P/6) [117]. Phenotypically these mice showed all the characteristics of aging and over the years there were several strains emerged that incorporated more of age-associated co-morbidities [118]. By 2001, cellular senescence was not considered as a mechanism for osteoporosis [119].

In fact, it was not until recently that senescent osteoblasts, osteocytes, and myeloid populations were identified during physiological aging [25] and in a pathological model of accelerated aging using focal radiation [61]. Markers of senescence p21, p16^Ink4a^, and p53 were identified not only in mice but in aged bones from human biopsies [25]. Targeted removal of senescent cells, either pharmacologically, using senolytic drugs or genetic clearance of p16-positive cells in *INK-ATTAC* mice, or by the targeted inhibition of Janus kinase pathway, which in turn blocked SASP production, alleviated age-related osteoporosis in mice [120]. p16-3MR mice, is another genetic model for the clearance of p16-positive cells. Clearance of p16-expressing cells failed to show any recovery in the age-related bone loss [121]. However, the p16-3MR mice was not a good model for clearance of senescent osteocytes [121] as seen in the *INK-ATTAC* mice [120]. Intriguingly, studies done withp16-3MR mice showed that the clearance of senescent osteoclast progenitors did not have any effect on the bone architecture of aged mice. The study claimed that cell senescence was still the key mechanism underlying age-associated osteoclastogenic potential of myeloid cells, with the key SASP factors regulating osteoclast function were released by senescent osteoblasts and osteocytes. These data suggest a direct role of senescent osteocytes in the pathophysiology of age-related osteoporosis. A genetically targeted clearance of senescent osteocytes may answer this question in future. In a model of high oxidative stress induced senescence, it was shown that countering senescent cells with senolytic drugs could alleviate radiation-induced skeletal aging like phenotypes [61].

### 4.1. Senescence Associated Secretory Phenotype (SASP)

Senescent cells have a unique secretome, known as senescence-associated secretory phenotype (SASP), pro-inflammatory in nature becoming one of the hallmarks of senescent cells. SASP proteins may have diverse functions, but the primary function is to recruit immune cells for the clearance of senescent cells [122]. When the senescent cells overwhelm the body as seen during aging, the impaired immune function fails to remove the senescent cells, resulting in a sustained SASP production causing systemic morbidity.

As discussed earlier, SASP production is dependent on ROS production and can distinguish quiescent cells from senescent cells. SASP proteins are produced in response to a DDR [123] and may comprise of proteins such as cytokines, chemokines, and interleukins. ROS induces a DSB, which triggers a DDR finally resulting in activation of NF-кB stimulating the SASP secretion. It was shown that activation of DDR induces the transcriptional upregulation of GATA4, which then activates NF-кB and elevated SASP gene activation [124]. The idea that a senescent cell is always associated with a SASP was questioned by the findings when studies showing senescent cells with *p16^Ink4a^* expression, were reported without a significant SASP [125]. Hence, production of SASP in a senescent cell relied on the presence of a DDR. Mechanistic target of rapamycin (mTOR) pathway has also been shown to play an important role in cellular senescence and aging [126,127], and the activation of p38/mTOR pathway is required for a sustained SASP production [128]. Glucocorticoids, such as corticosterone and cortisol were shown to suppress the SASP production without the reversal of the senescent state of the cell [129].

SASP factors affecting skeletal aging were recently identified in enriched osteoprogenitors (characterized by Lin-/Lepr+), osteoblasts (characterized by AP+/CD1/34/4/54-), osteocytes (digested vertebra) and bone marrow myeloid cells (CD14+) (Figure 1) [25,130]. The majority of SASP factors were produced by the bone marrow myeloid cells and osteocytes, while only a small subset was expressed in osteoblasts and osteoprogenitors. Production of SASP during physiological skeletal aging shares some common features with pathological skeletal aging [25] such as that seen with radiation [61]. Suppression of SASP using Janus kinase inhibitors (JAKi) alleviated age-related bone loss [120]. A better understanding of heterogeneity of SASP production was seen in an enriched population among different bone cells in mice, with varied expression levels, with larger fold changes seen in myeloid cells of aged bones, as compared to aged osteocytes (Figure 1) [25]. These results were largely replicated in human bone biopsies [25] and radiated mouse bones [61]. In another instance increasing doses of radiation induced proportional levels of senescence and gene expression for SASP markers in rat BMSCs [131]. Lipopolysaccharides have also been shown to induce senescence in alveolar bone together with the SASP factors such as Icam1, Il6, Mmp13, and TNF-alpha [132]. However, with a better understanding of senescence as a driver of age-related osteoporosis, but not post-menopausal osteoporosis [4], the correlation between senescence and bone loss in general is not a linear relationship. The SASP profile in the bones of mice which have undergone either orchidectomy or ovariectomy in young mice did not have resemblance with aged bones and remained mostly non-significant. Similar results were obtained with *INK-ATTAC* mice, in which ovariectomy induced bone loss was not recovered post-clearance of *p16*-positive senescent cells, and clearance of senescent cells did not have any effect on senescence markers. However, a short-term estrogen treatment could suppress age-related senescence and SASP markers [4], suggesting that estrogen may regulate senescence-pathways during old age. Since DDR is a key factor in SASP production, there may be several kinds of pathological osteoporosis where SASP is different from age-related osteoporosis. It was recently shown that ATM, other DDR proteins and NF-кB pathways were greatly elevated in Ercc1 deficient mice, in which the NER pathway of DDR was affected. These mice had a higher senescence and SASP profile which was reduced following the suppression of ATM kinase [133]. These studies suggested that targeting ATM pathway could slow the progression of aging, however there are contradictory studies as well where ATM activation alleviates senescence [134]. Moreover, histone variant macroH2A1, an epigenetic modified form of the canonical H2A histone and a marker for SAHF, is one of the recent additions to the proteins that in response to oncogene activation, may regulate SASP production and a persistent DDR, controlled by both positive and negative feedback loops [135]. Variants of macroH2A1, macroH2A1.1, and macroH2A1.2 increase with old age [136]. While a lot has not been reported on the role of macroH2A in bone homeostasis, macroH2A1.2 has been shown to negatively regulate breast cancer-induced osteoclastogenesis, by cooperating with Ezh2 [137]. Interactions between macroH2A1.1 and PARP1 regulate mitochondrial activity and a stress response, which can then regulate the SASP production, an area open for further exploration.

### 4.2. PARP1: Role in Senescence and Skeletal Aging

PARP1 belongs to a family of transferases which is localized in the nucleus and is an important DNA damage response (DDR) protein. Association of PARP1 with DNA repair process [138] and telomere maintenance [139,140] push the researchers to find the evidence of its role in longevity. PARP1 is known to be a general caretaker of the genome as it participates in major repair pathways and can be called as a first responder DDR protein. Several in vivo studies have supported the role of PARP1 in longevity. Telomeric DNA was approximately reduced by 30% in PARP knockout mice [141] as also observed with PARP knockdown or inhibition in cell culture [140]. This regulation of telomere length by PARP1 at molecular level is due to interaction with telomeric repeat binding factor 2 (TRF2) and thus affecting its binding to telomeres [139,142]. PARP1 modifies target proteins by covalently linking PAR (poly(adenosinediphosphate-ribose)) moieties, a post-translational modification process known as PolyADP-ribosylation or PARylation. PARylation status among 13 mammalian species strongly correlated with their maximum life span, wherein, PARylation was found to be five times higher in PBMCs of humans as compared to rodents [143]. Furthermore, PARylation levels in PBMCs were reported to decline with age [144]. Intriguingly, centenarian humans showed higher PARP activity than the young subjects [145,146]. Dynamics of PARP activity also changes with restriction of cell proliferation which leads to accumulation of age-related macromolecular changes including DNA [147]. Human-PARP1 overexpressed mice had prolonged disease-free survival, reduced tumor burden, but were more susceptible to aging related metabolic disorders. This has raised a question whether PARP1 is the probable candidate for longevity.

In addition, PARP1 is reported to play a role in inflammation [148] and caspase independent cell death [149,150], hence could act as an aging promoting factor. PARP1 is known to be a transcriptional coactivator of NFкB [151], which is an important mediator of inflammatory signaling and aging [152,153]. Severe DNA damage and NFкB directed inflammation could hyperactivate PARP1 that leads to necrosis due to depletion of NAD and ATP pool of a cell [154]. PARP1 dependent pathologies to some extent accumulate and lead to neurodegenerative disorders and aging. Therefore, PARP1 acts as a double-edged sword, where it acts as a longevity factor as well as an age promoting factor. PARP1 is an interesting player which exhibits contrasting roles in cell.

PARP1 has an inverse relationship with SIRT1, a longevity associated enzyme belonging to the sirtuin family (NAD dependent deacetylases). PARP activity limits the bioavailability of NAD for SIRT1 activity, and henceforth reduces the deacetylation of certain transcriptional factors including PGC1α which would affect mitochondrial biogenesis and ultimately aging [155]. Recent work by Zha and colleagues, 2018, proposes the use of PARP inhibitors to maintain mitochondrial function and function of aging induced endothelial progenitor cells (EPCs) by SIRT1 activation [156]. These findings suggest PARP1 as a longevity regulator where it can be a positive or negative regulator in a context dependent manner. There is a need to recognize the scenarios where PARP activity balances genomic integrity and metabolism to regulate aging.

#### 4.2.1. PARP1 in Senescence

Persistent DNA damage stimulates senescence in cells and PARP1 being a DNA repair enzyme do play a role in cellular senescence. A major non histone chromatin component, DEK protein has a role to play in metabolism and DNA repair. Increased DEK levels are known to favor immortalization by impeding senescence and apoptosis, while DEK deficient cells during genotoxic stress induces senescence [157]. Moreover, DEK is PARylated by PARP1 and hence regulates its activity in response to genotoxic stress [158]. Interestingly PARP1 inhibition increased the cellular senescence, while p21 deletion enhances PARP1 activity and DNA repair by NHEJ, thereby reduces DNA damage and subsequently cellular senescence [159]. PARP1 is a new target for treating various tumors and some studies have elucidated the role of PARP inhibitors in senescence. In ovarian cancer cells, low dose administration of olaparib has induced cellular senescence rather than apoptosis. The study suggested that olaparib induces senescence via p16-Rb or p53-Rb signaling axis and thereby inhibited the cell proliferation [160]. Intriguingly, PARP1 and its family members play key roles in regulating the SASP factors, cytokines, and metalloproteases. PARP1 is reported to be associated with the promoters of cytokines, TNFα and IL1β [161]. Histone variant macroH2A1 plays a crucial role in regulating certain SASP genes at transcriptional level. Further, macroH2A1.1 is reported to regulate PARP1 activity either by recruiting it to chromatin [162], and hence could mediate SASP gene expression through PARP1 [135]. As already discussed above, PARP1 regulates mitochondrial function and metabolism; hence, macroH2A1 and PARP1 axis could play a key role in senescence and aging which needs to be investigated. Thus, there is a high probability of PARP1 contributing to senescence and its associated phenotypes.

#### 4.2.2. PARP1 Role in Metabolism and Effects on Cellular Aging

Metabolism is considered to slow down with age, whereby metabolic abnormalities are key hallmarks of aging. Dietary restriction (DR) is testified to extend the lifespan of an organism, and thus could affect the longevity and good health in humans, but further research is required to prove the DR effects keeping in criteria the early or late onset of DR [163]. Various research has linked PARP1 with the aging associated metabolic diseases [164,165,166] as well as brain diseases [167]. PARP1 is known to affect metabolism either directly or indirectly, wherein, PARP activation limits the metabolic fitness of a cell. PAR signaling could affect the activity of enzymes like hexokinase and hence glycolysis [168,169]. Moreover, PARP utilizes NAD a critical metabolic cofactor, thus hinders cellular energy production [170]. PARP activation and NAD consumption in response to DNA damage sometimes shift the metabolism from oxphos to glycolysis resulting in damaged cell survival [171]. Recent preclinical results have highlighted the role of NAD metabolism in aging and hence restoration of NAD levels in old animals could extend lifespan and promote good health [172]. Researchers are exploring ways to boost NAD levels in cell to attain healthy aging and longevity. NAD supplementation, activation of NAD biosynthetic enzymes, and inhibition of NAD degrading enzymes are three main approaches to increase NAD levels. Sirtuins and PARPs are two major NAD consuming enzymes, and hence targeting them would be a beneficial strategy in aging. In this context, inhibition of the PARP1 enzyme would prevent degradation of NAD and would thus maintain NAD levels in cells and further delay in aging.

In addition to genomic instability, mitochondrial dysfunction is another key player in cellular aging. Mitochondrial DNA (mtDNA) mutations originate either due to oxidative stress or as replication errors by the mitochondrial DNA polymerase. Such mtDNA mutations thereby contribute to age associated diseases and aging phenotypes [173]. In addition, nuclear DNA damage initiates nucleus to mitochondrial signaling which may regulate mitochondria function and aging. This signaling network involves nuclear sirtuins and PARPs that regulate genomic stability as well as mitochondrial integrity [174]. Elucidation of the PARP signaling and mitochondrial function relationship would provide a new direction to research on aging. PARP, when hyperactivated, was shown to affect metabolism and mediate cell death and senescence [175]. In vitro and in vivo inhibition of PARP1 boosted NAD levels, enhanced SIRT1 activity, mitochondrial content, and augmented oxidative metabolism [164]. It will be enlightening to study how PARP connects to mitochondrial function and mitophagy in the aging process.

#### 4.2.3. PARP1 Role in Skeletal Aging

ADP ribosylation (PARylation) is proposed to regulate the differentiation of bone cells, and hence has an impact on bone health. PARP1 has been shown to regulate osteoclastogenesis [176] and osteogenic differentiation [177]. Accumulation of PARP1 leads to biomineralization of bone and vasculature triggered by a DDR, leading to excessive extracellular matrix calcification [178], also associated with senescence [179]. Vascular calcification and bone loss are major disorders associated with aging. Bone mineral density and vascular calcification has an inverse relationship seen specifically in women, but not men [180]. PARP1 expression [181] and activity [182] has been found to increase in calcified aortic valves and vascular smooth muscle cells, respectively. PAR moieties have high affinity for calcium and thus assist in bone mineralization [178]. Although there is no direct evidence of PARP1 in skeletal/bone aging, but its role cannot be neglected keeping in view its involvement in bone development and homeostasis. Further work is required to identify the connection between PARP1 and bone aging.

## 5. Therapeutics for Aging Bone

### 5.1. Parathyroid Hormone (PTH)

Parathyroid hormone (1-84amino acid; PTH) is an important regulator of calcium homeostasis, where the blood calcium level is controlled by the release of calcium from the existing bone, by a calculated action of osteoblasts over the osteoclasts. PTH is one of the first hormones whose efficacy was considered for the treatment of senile osteoporosis. PTH 1-34 (Teriparatide; Forteo^®^) is a biosynthetic drug composed of the first 34 amino acids of human parathyroid hormone. It was one of the first anabolic drugs approved for osteoporosis in the European Union and in the US by the FDA [183,184,185]. Intermittent teriparatide treatment is prescribed for patients who are at high fracture risk. It is currently approved as an injectable and is very effective in improving the overall BMD. The anabolic effect of teriparatide is not fully understood and while it has been shown to improve osteoblast function, increase osteoblast formation and decrease in osteoblast apoptosis, the exact mechanism of the conversion of the progenitors in osteoblasts, role of blood vessels and the movement of cells during bone formation is still under investigation. The use of teriparatide as a treatment of osteoporosis is limited for two years, a limitation assigned based on the high rate of occurrence of osteosarcoma in animal studies [186]. However, long-term follow up in humans, have not reported a single case of osteosarcoma in patients who have received teriparatide treatment [187].

Several studies including ours have investigated the efficacy of teriparatide to counter triggers of senescence by promoting DNA repair mainly through the activation of the Wnt pathway [55,188]. PTH administration was shown to downregulate senescence by inhibiting p16^Ink4a^ and alleviated the age-related progression of osteoarthritis [189].

### 5.2. Anti-Sclerostin Antibody

Sclerostin, a glycoprotein encoded by the gene SOST, is secreted by osteocytes, has inhibitory effects on the osteoblast function by negatively regulating the Wnt and bone morphogenetic protein (BMP) signaling. Sclerostin has been shown to negatively regulate several cellular processes in the bone. Sclerostin levels in serum and at mRNA level has often been used as a predictor of bone health. High serum sclerostin was found to be associated with occurrence in osteoporotic fractures in post-menopausal women [190,191]. Interestingly intermittent treatment of PTH could suppress the serum sclerostin levels [192]. In one of the earliest assessments of serum sclerostin in aged men and women, serum sclerostin was found higher in men than in women, and sclerostin levels were significantly higher in elderly individual as compared to their younger counterparts [193]. In similar studies sclerostin levels were found to be elevated in elderly people [194,195]. In another study serum sclerostin was elevated only in aged-men, but not women [196]. A Genome wide association study (GWAS) done in a small subset of post-menopausal Norwegian women, serum sclerostin and mRNA levels of sclerostin were found to be reduced, while detecting elevated level of *SOST* promoter to be methylated [197]. These changes were attributed to the lower number of osteocytes producing sclerostin. The varying levels of sclerostin reported in different studies involving age and sex differences should be taken in context with the exercise regimen and the level of load bearing exercises performed by those individuals, as exercise has been shown to reduce sclerostin levels, even in post-menopausal women [198,199]. A significant decline in mobility is expected in elderly people, leading to high serum sclerostin levels.

Similar level of elevation in sclerostin levels is observed in osteoclasts from aged mice [200]. Elevated levels of sclerostin were also reported in radiated bones, another model of skeletal aging [56]. It was shown that sclerostin may be responsible in generating radiation-induced DNA damage, since use of a neutralizing antibody against sclerostin promoted DNA repair, suppressed radiation-induced adverse changes in bone marrow including adiposity and alleviated loss in bone architecture due to radiation damage [56]. Interestingly, we also reported that following oxidative stress caused by ionizing radiation, sclerostin mRNA did not change, but histological evidence proved that sclerostin protein was elevated several folds [56].

A humanized antibody against Sclerostin (romosozumab) is an emerging therapeutic, which has now been approved by FDA for treatment under the brand name “Evenity” (romosozumab-aqqg in the US). The limitation of the Sclerostin action within the skeleton makes it a good candidate for osteoporosis, with fewer concerns of systemic effects. Sclerostin’s anabolic function and as a possible therapeutic for osteoporosis, is based on the high bone mass phenotype in patients of sclerosteosis with a genetic deficiency of sclerostin [201,202]. Similarly, a genetic deletion of sclerostin in mice also resulted in a high bone mass [203]. A pre-clinical study in rats confirmed the efficacy of anti-sclerostin antibody in a model of postmenopausal osteoporosis [204]. In large scale clinical trials, it was reported that romosozumab was associated with an increase in BMD [205], with a better anabolic effect than teriparatide [206]. Unlike teriparatide, romosozumab had no carcinogenicity concerns in animals or humans [207]. Effects of romosozumab were reversible when discontinued and required a subsequent treatment of denosumab, an antiresorptive monoclonal antibody against RANKL [208] and by a single dose of zoledronate, which preserved the anabolic bone accrual initiated by romosozumab, for an additional two years [209].

### 5.3. Anti-Resorptives

The class of drugs that suppress the osteoclast-based bone resorption are termed as “anti-resorptives”. Bisphosphonates are a class of anti-resorptives which have a pyrophosphate-like chemical structure which allows them to bind strongly to calcium and may work as a beacon to the bone tissue. The nitrogen-containing bisphosphonates which are not limited to etidronate, clodronate, risedronate, alendronate, olpadronate, ibandronate, and zoledronate [210,211]. These nitrogen-based bisphosphonates target farnesyl diphosphate synthase (FPP synthase), an enzyme in the mevalonate pathway, thereby suppressing the osteoclast function. Zoledronate has become one of the most widely accepted anti-resorptive and as a treatment for osteoporosis. It was recently reported that zoledronate can improve DNA repair in MSCs [54], suggesting that it may work as regulator of cell senescence and hence be used as a therapeutic for skeletal aging, which is markedly different from post-menopausal osteoporosis. Denosumab, a monoclonal antibody against RANKL, blocks binding of RANKL to its receptor RANK on the osteoclast progenitors, suppresses osteoclast function, and thus protects bone loss as an anti-resorptive. While denosumab may work to alleviate senile osteoporosis, but there is no evidence to suggest that it may have a role in regulating senescence as a mechanism.

### 5.4. Senolytics and SASP Modulators

Identification of senotherapeutic drugs were based on the compounds that would selectively kill senescent cells without affecting proliferating cell termed senolytic drugs, or drugs that suppressed cell senescence or SASP termed senomorphic, while some other compounds that are toxic to cells, have no effect on senescent cells, increase senescent cells or increase in proliferation, were excluded as senotherapeutics [212].

Since the identification of senescent osteoblasts and osteocytes in bone tissue, it was speculated that the senescent nature of these cells together with the SASP regulated bone remodeling. Clearance of senescent cells and suppression of SASP hence became lucrative methodologies to treat physiological and pathological skeletal aging. Genetic clearance of *p16^Ink4a^* has been shown to improve age related health and life span [47], and age-related osteoporosis [120]. Pharmacological clearance of senescent cells using a senolytic drug cocktail of Dasatinib and Quercetin was also effective in restoring bone architecture as seen in physiological aging [120] and in a pathological model of skeletal aging as seen with radiation-related osteoporosis [61]. However, some other senolytic drugs which were shown to be effective in curing some aspects of age-related comorbidities, were ineffective in radiation-associated bone loss [61], suggesting varied mechanisms of actions among the senolytic drugs. Clinical trials are currently underway to assess the efficacy of senolytic drugs to treat age-related comorbidities, including osteoporosis.

Hence, while anabolic agents can promote bone formation, and anti-resorptives can suppress osteoclast function, senolytic drugs can eliminate the senescent cells responsible for instigating osteoclast activity and suppression of bone formation, making senolytic drugs as a promising treatment strategy for age-related osteoporosis.

Several novel drugs have been explored as senolytic, including Dasatinib (D), Quercetin (Q), D+Q [213], Luteolin [214], Fisetin [215,216], Navitoclax (ABT263) [217], BCL-XL inhibitors [216], HSP90 inhibitors [212] Piperlongumine [218], RG7112 [219], O-Vanillin [219], ABT-737 [220], and CD153 Vaccine [221] and aspirin [222,223], reviewed by Robbins et al. recently [224] (Figure 2).

Drugs that do not kill the senescent cells but counter the pro-inflammatory protein production are termed as SASP modulators or senomorphics. JAKi, ruxolitinib have been shown to be effective in alleviating age-related osteoporosis, by possible suppression of specific factors such as IL6, IL8 and PAI1, which were shown to activate osteoclast formation [120]. Inhibitors for the Mdm2 can block the interaction between Mdm2 and p53 and block p53 degradation, hence can lead to high p53 and p21 expression. However, the same Mdm2 inhibitors, Nutlin3a and MI-63, have been shown to suppress SASP factors [225]. Rapamycin and Rapalogs (analogs of Rapamycin) are also reported to suppress the SASP [226,227].

## 6. Future Directions

With the increase in identification of several target molecules that positively or negatively regulate the bone, advent of new and more effective therapeutics is inevitable. These newer therapeutics should have minimal side effects and their efficacy during other disease conditions should also be explored. So, while the patient is treated for osteoporosis, the drug should not interfere with the function of the drug for a secondary disease. This could be achieved by using single, alternate, or combinatorial treatment and determining the efficacy or toxicity of both or either of the drugs. Alternatively bone anabolic agents could be fused with a “homing” molecule which would guide the drugs only to the bone surface, minimizing the systemic effects on other organs. An osteoblast specific loss of RICTOR, an mTOR complex2 protein resulted in age-related bone loss [228]. Sirt-3, an important protein in mitochondrial metabolism, activates the mTOR pathway to regulate osteoclastogenesis, increased adipogenesis and bone loss [229]. The role rapamycin and similar mTOR inhibitors as senolytic or senomorphic has not been studied in the context of an aging skeleton, but rapamycin was able to alleviate periodontal diseases in aged mice [230].

PARP1 is another interesting DDR protein, which not only plays a role in DDR, but also plays a role in inflammation by regulation of the NF-кB pathway. As seen with ATM kinase levels, PARP1 levels beyond a certain threshold may be detrimental for certain age-related co-morbidities. One such example is where PARP1 inhibition initiated prevention of neurodegeneration seen during Parkinson’s disease, by restoring degradation of alpha-synuclein [231]. There are other examples where PARP1 inhibition alleviated age-related cellular [156] and tissue dysfunction [232] and could be a potential therapeutic option for osteoporosis.

As discussed above, age-related increase in cellular senescence and the proinflammatory SASP drives several age-related comorbidities including osteoporosis. So, clearance of senescent cells is a lucrative option. This is currently being tested in several clinical trials including patients with diabetic kidney disease (ClinicalTrials.gov Identifier: NCT02848131), idiopathic pulmonary fibrosis (ClinicalTrials.gov identifier: NCT02874989), and age-related osteoporosis (NCT04313634). Some of the novel senotherapeutic drugs which have shown efficacy in vitro, may be tested as a treatment of osteoporosis. However, judgement should always side with caution, since senolytic drugs may work for recovering some aged-related tissue dysfunction [217], but not for osteoporosis, as in the case with Navitoclax/ABT-263 [233]. Another example is Fisetin which also worked as a senolytic and alleviated several age-related phenotypes [215,216], but not skeletal aging seen with radiation exposure [61]. Fisetin may work for resorption-based osteoporotic diseases as it counters the osteoclast function [234,235], but its role in age-related osteoporosis is yet to be determined.

Future treatments of osteoporosis and other bone ailments may include senotherapeutics which may be explored to be given in combination with the more established bone anabolic drugs.
